# Supplementary material for: In-conduit capture of sub-micron volcanic ash particles via turbophoresis and sintering
Source: Nat Commun. 2022 Aug 11;13:4713. doi: 10.1038/s41467-022-32522-7 (PMC9372141; doi:10.1038/s41467-022-32522-7)
Supplement: Supplementary file 1 — Supplementary Information [file 41467_2022_32522_MOESM1_ESM.pdf]

Supplementary information

In-conduit capture of sub-micron volcanic ash particles via turbophoresis and sintering

Jamie I. Farquharson[1], Hugh Tuffen[2], Fabian B. Wadsworth[3], Jonathan M. Castro[4], Holly Unwin[2], C. Ian Schipper[5]

[1]Institut Terre Et Environnement de Strasbourg, UMR 7063, Université de Strasbourg, CNRS, 5 rue René Descartes, 67084, Strasbourg, France

[2]Lancaster Environment Centre, Lancaster University, Lancaster, LA1 4YQ, UK

[3]Department of Earth Sciences, Durham University, Durham, DH1 3LE, UK

[4]Institute of Geosciences, Johannes Gutenberg Universität, Mainz, D-55128, Germany

[5]School of Geography, Environment and Earth Sciences, Victoria University of Wellington, Wellington 6012, NZ

Supplementary Figure 1 | Scanning Electron Microscope (SEM) images from which grainsize distributions were measured. Sample names are noted on associated images: AN1, AN2, CCTVAIP, CCVP, and CC1960. Lines are overlain on the semimajor axis of measured grains.

Supplementary Figures 2–5 | Energy Dispersive X-ray (EDX) element maps. First panel shows layered image, subsequent panels are broken down by element.

Supplementary Figure 1 | Scanning Electron Microscope (SEM) images from which grainsize distributions were measured.

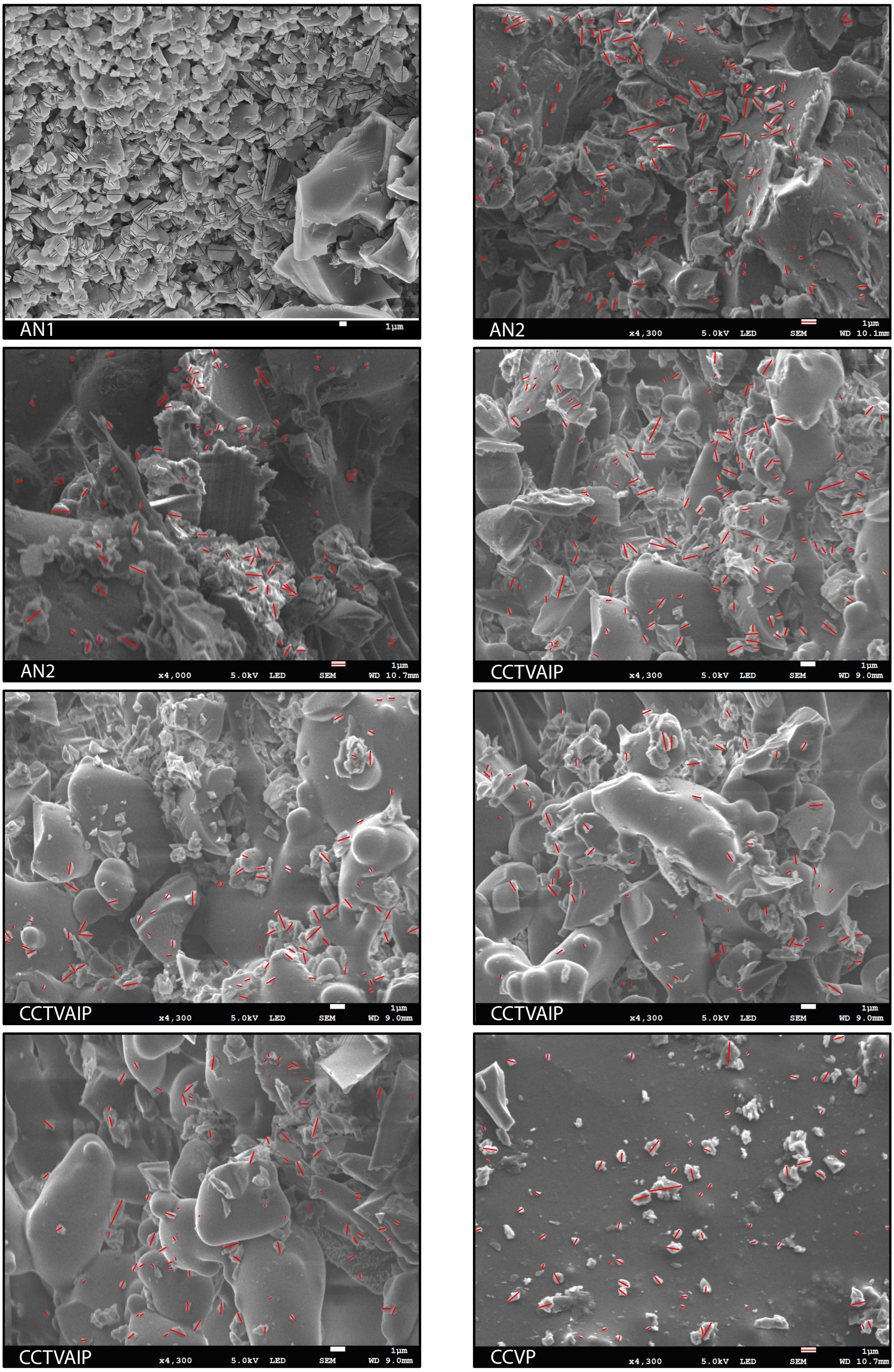

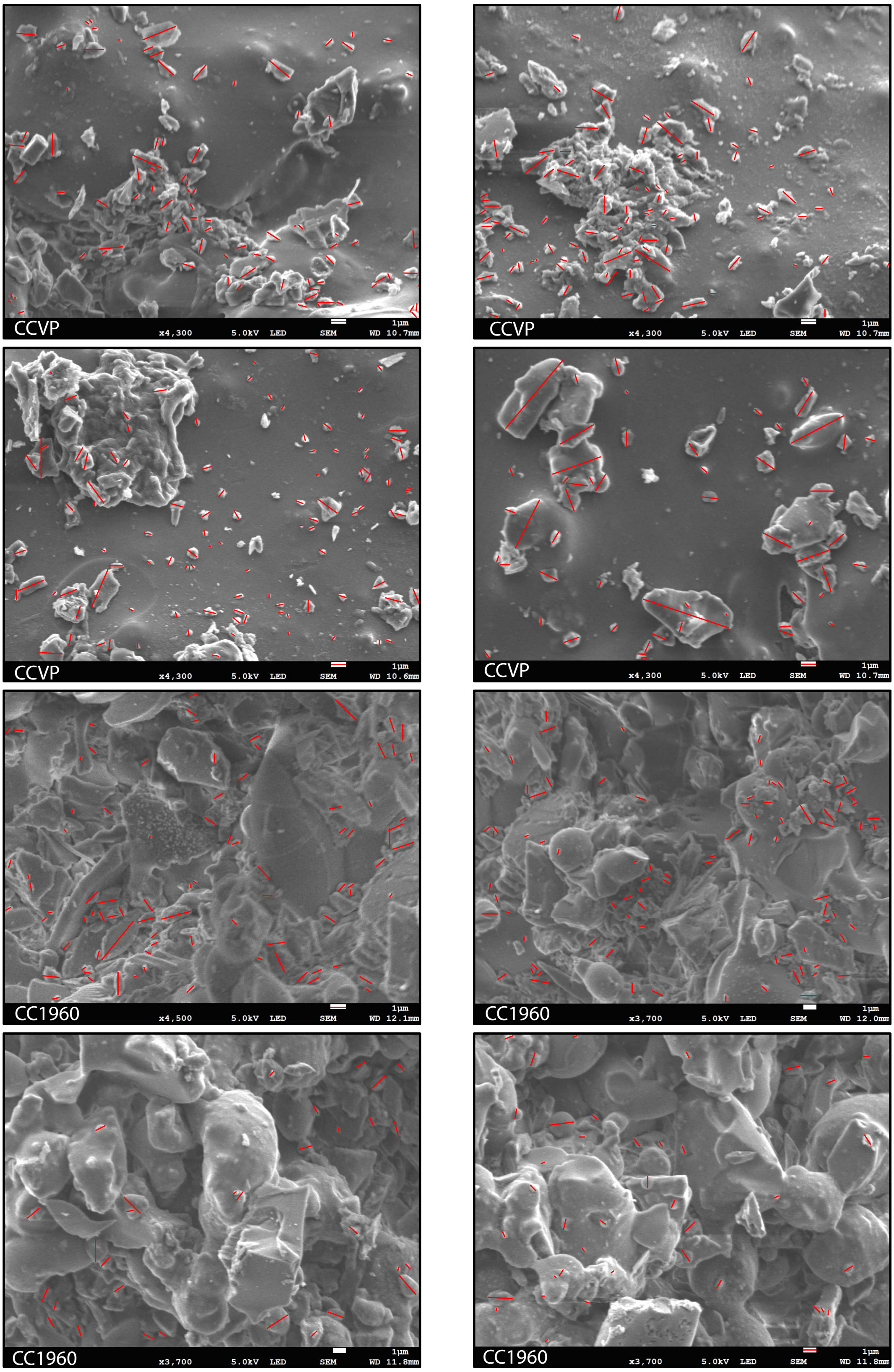
CCVP
x4,300 5.0kV LED SEM 1μm WD 10.7mm
CCVP
x4,300 5.0kV LED SEM 1μm WD 10.7mm
CCVP
x4,300 5.0kV LED SEM 1μm WD 10.6mm
CCVP
x4,300 5.0kV LED SEM 1μm WD 10.7mm
CC1960
x4,500 5.0kV LED SEM 1μm WD 12.1mm
CC1960
x3,700 5.0kV LED SEM 1μm WD 12.0mm
CC1960
x3,700 5.0kV LED SEM 1μm WD 11.8mm
CC1960
x3,700 5.0kV LED SEM 1μm WD 11.8mm

Supplementary Figure 2 | Energy Dispersive X-ray (EDX) element maps for sample CCTVAIP. First panel shows layered image, subsequent panels are broken down by element.

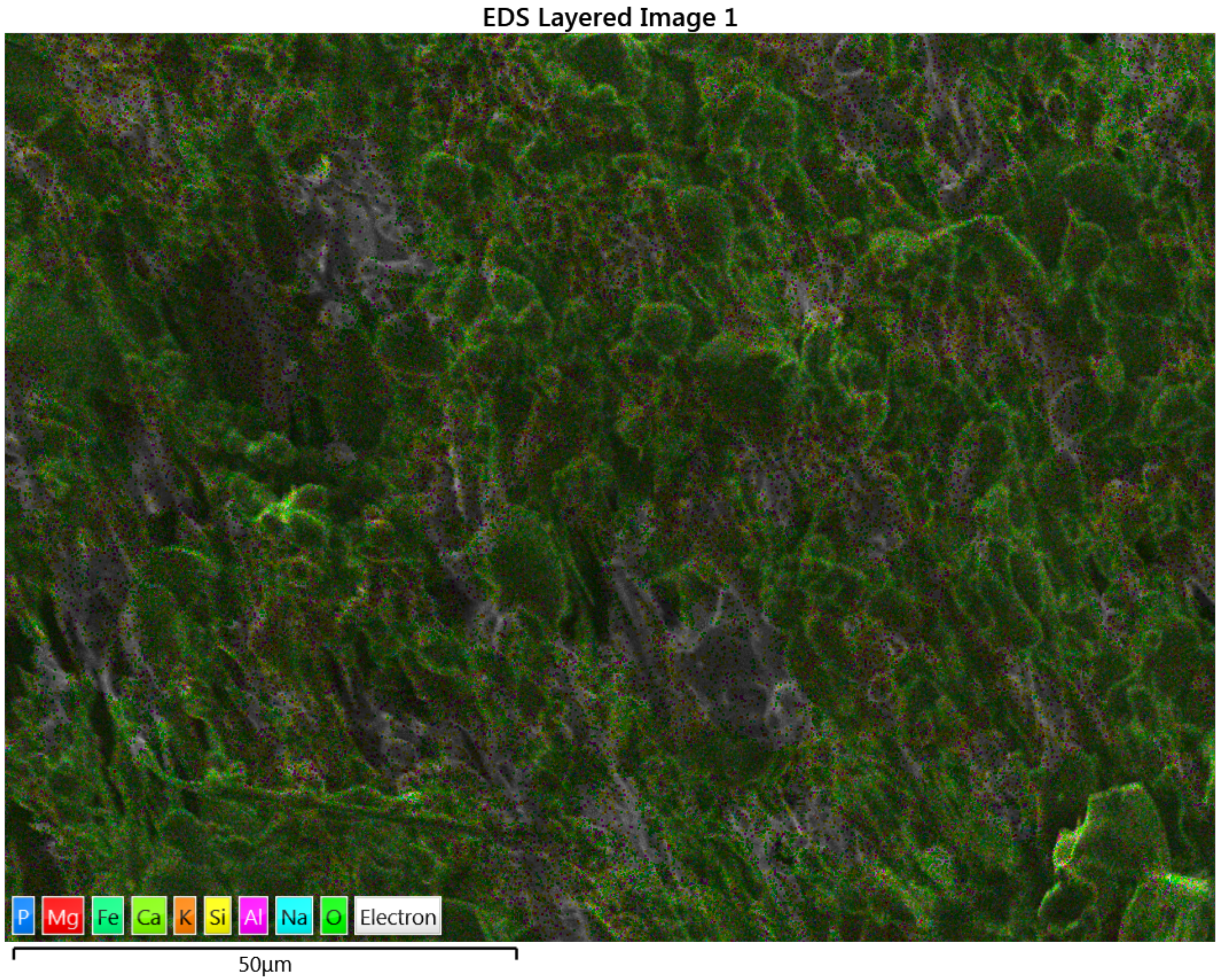

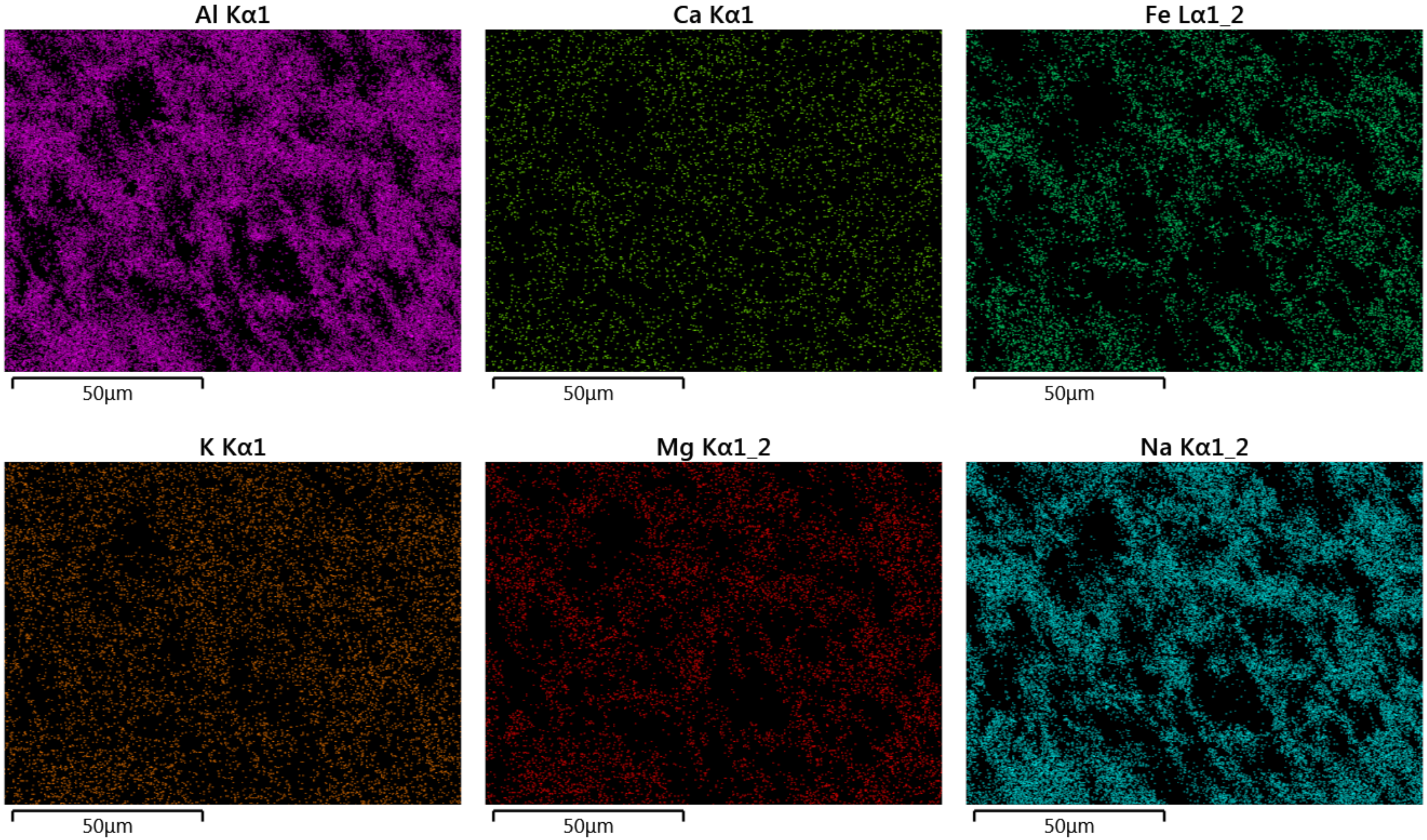
Al Kα1
Ca Kα1
Fe Lα1_2
K Kα1
Mg Kα1_2
Na Kα1_2
50µm

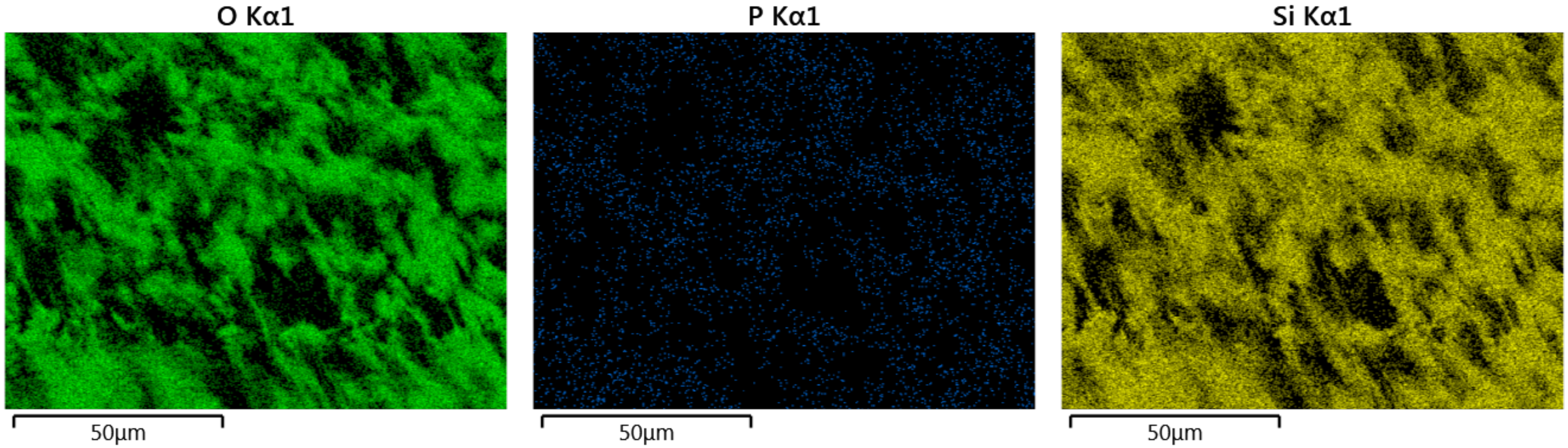
O Kα1
P Kα1
Si Kα1
50µm
50µm
50µm

Supplementary Figure 3 | Energy Dispersive X-ray (EDX) element maps for sample CCTVAIP. First panel shows Si only, subsequent panels are broken down by element.

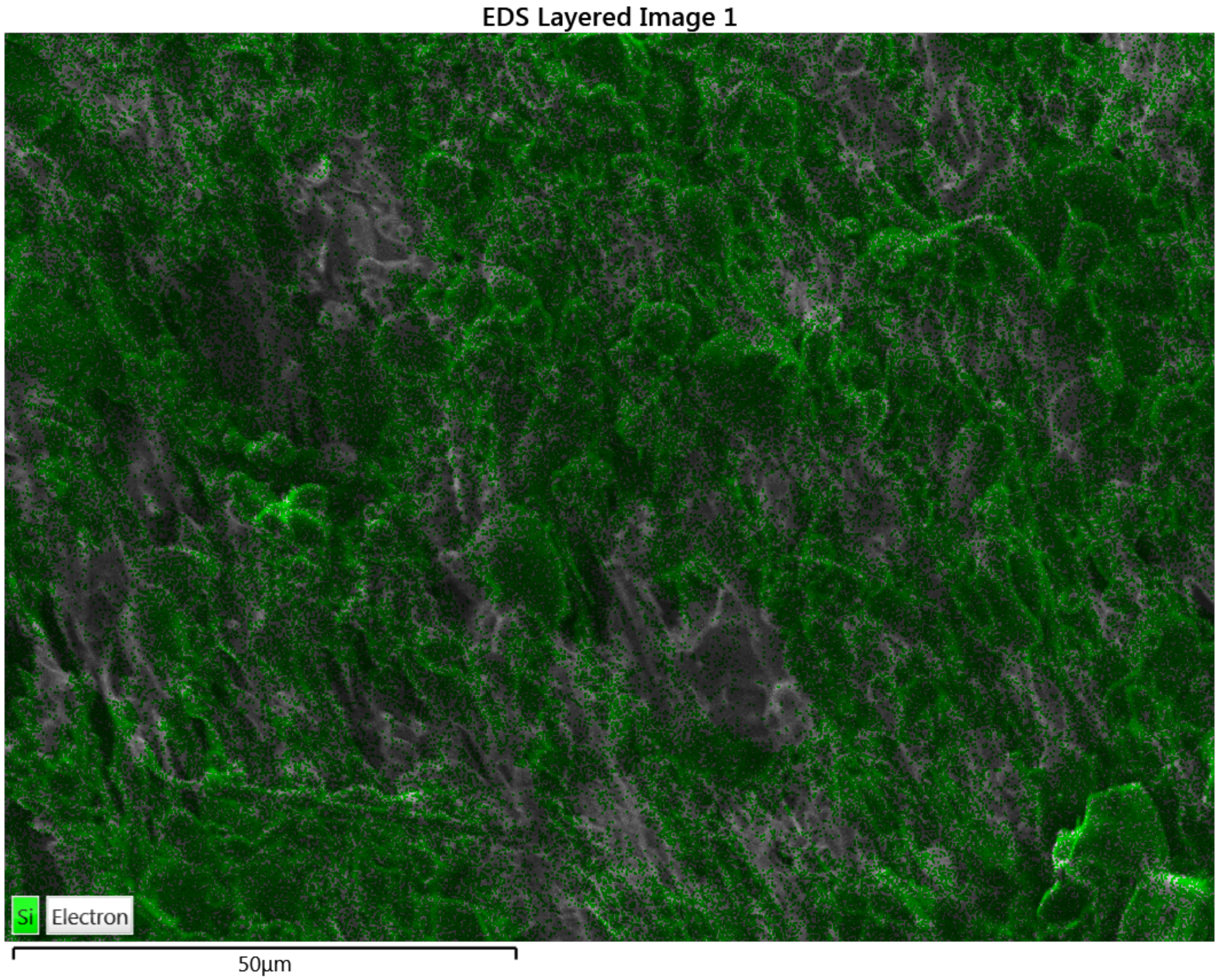

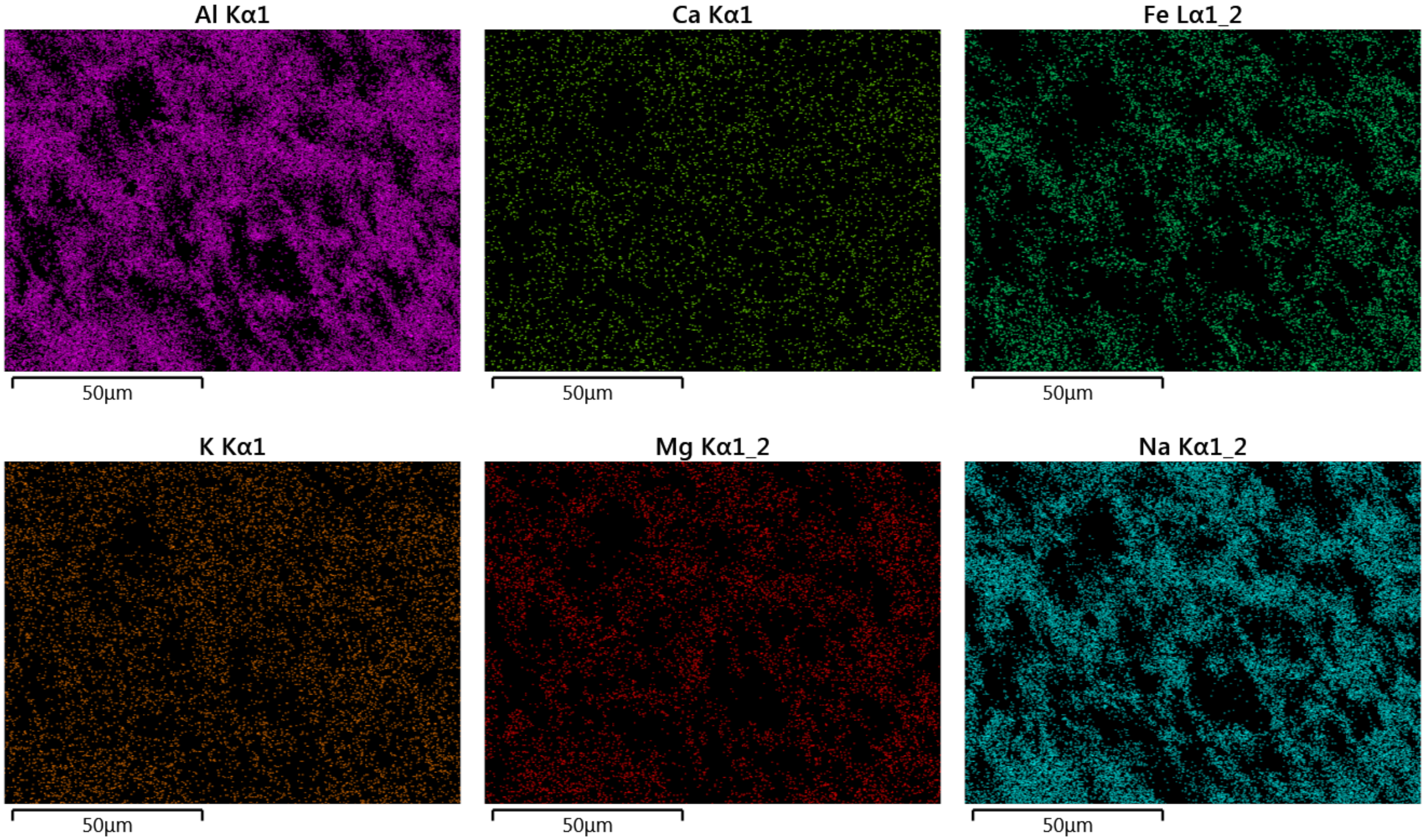
Al Kα1
50µm
Ca Kα1
50µm
Fe Lα1_2
50µm
K Kα1
50µm
Mg Kα1_2
50µm
Na Kα1_2
50µm

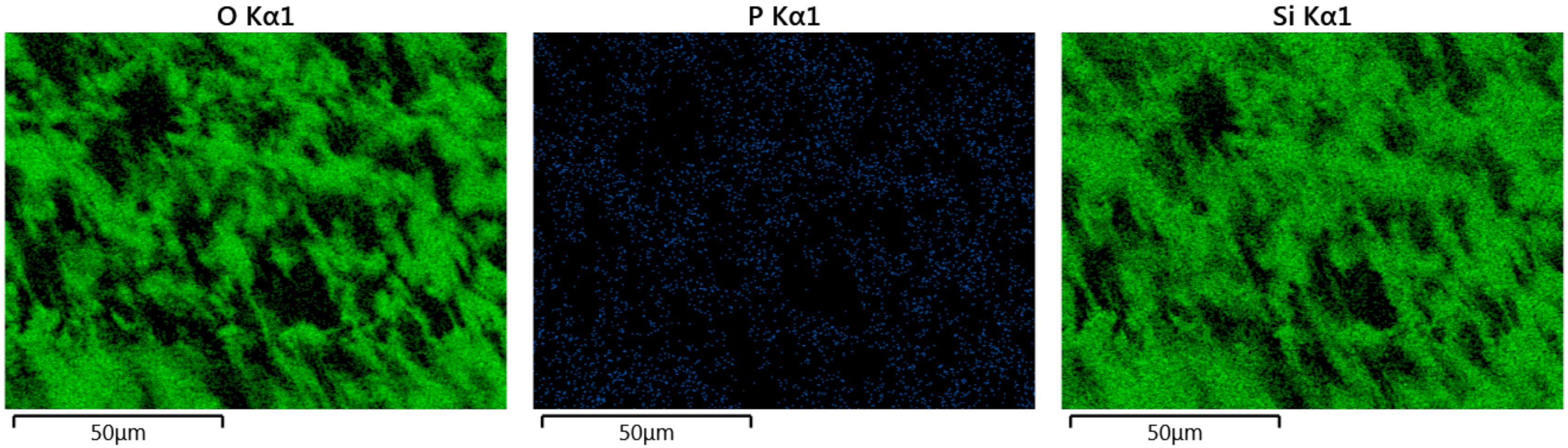
O Kα1
50μm
P Kα1
50μm
Si Kα1
50μm

Supplementary Figure 4 | Energy Dispersive X-ray (EDX) element maps for sample CCTVAIP. First panel shows layered image, subsequent panels are broken down by element.

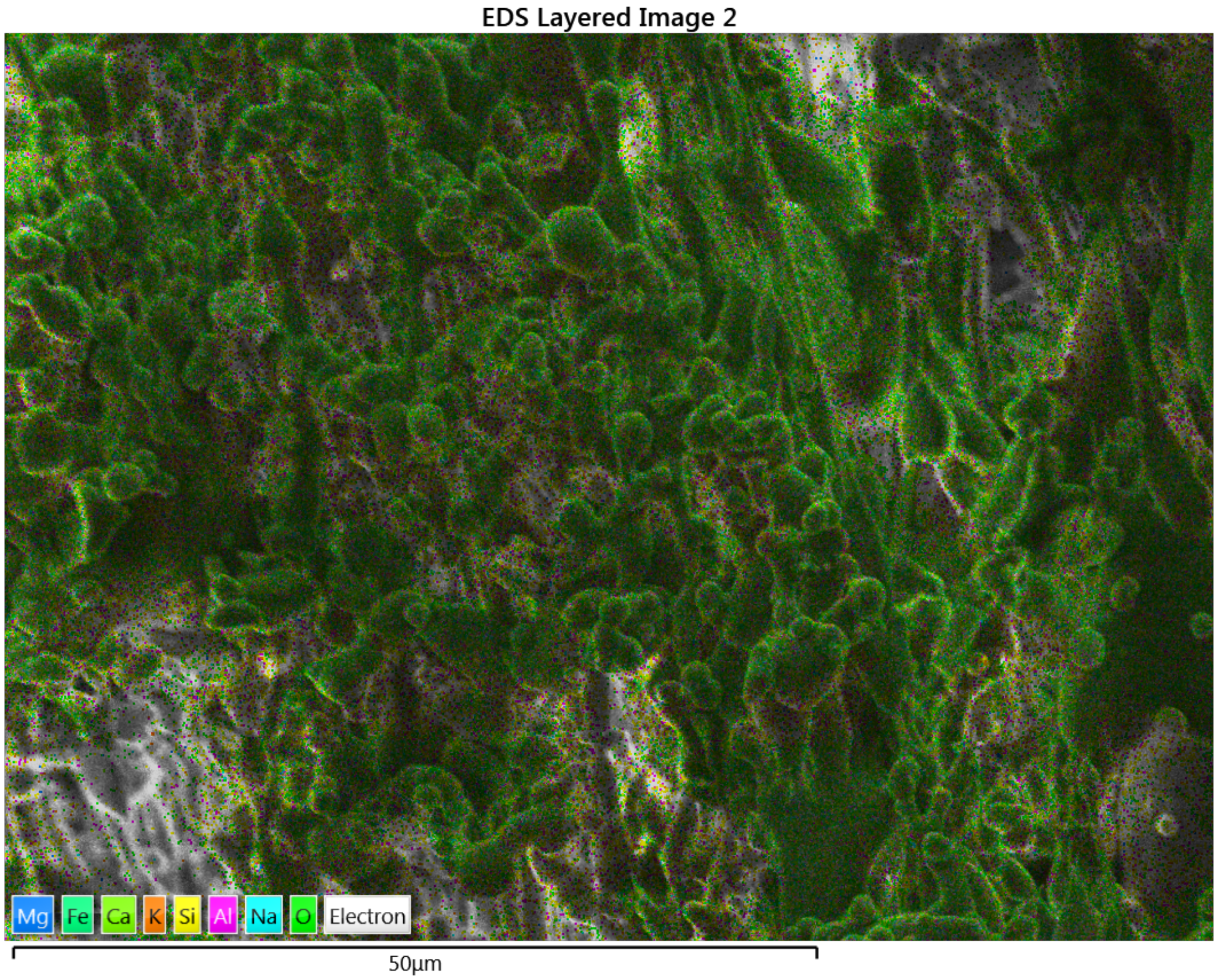

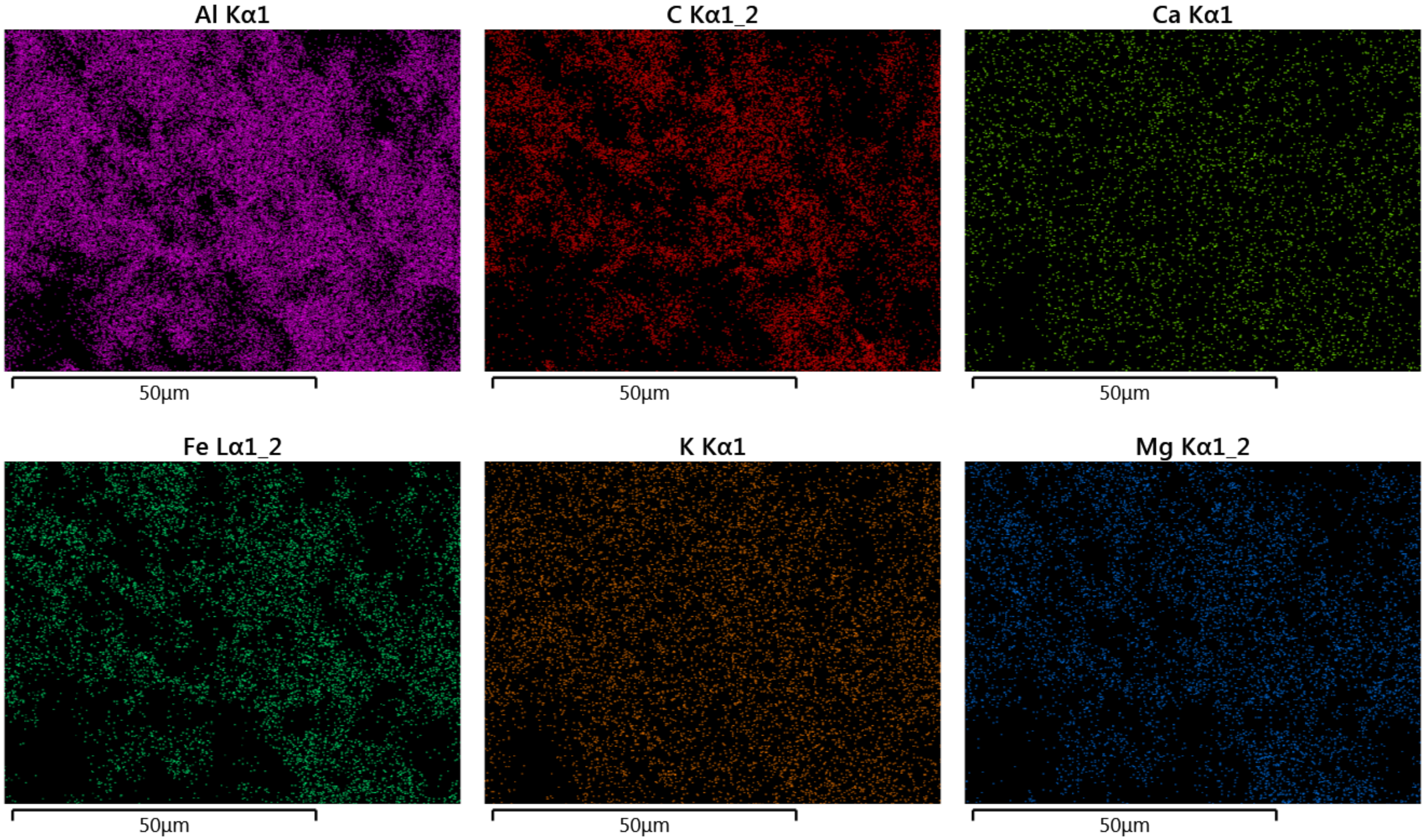
Al Kα1
C Kα1_2
Ca Kα1
50µm
50µm
50µm
Fe Lα1_2
K Kα1
Mg Kα1_2
50µm
50µm
50µm

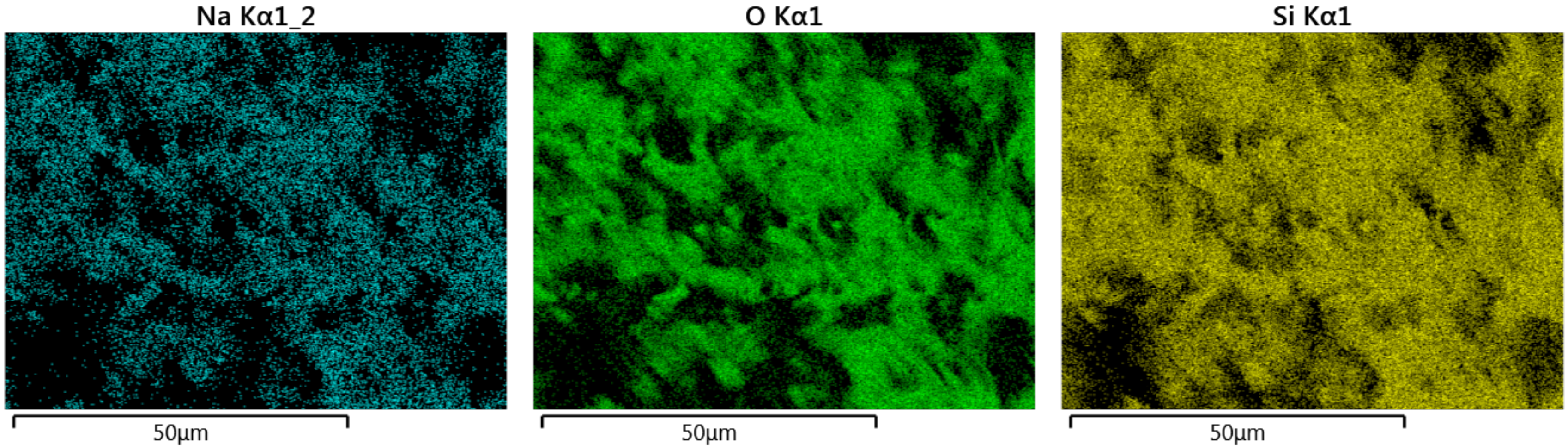
Na Kα1_2
O Kα1
Si Kα1
50µm
50µm
50µm

Supplementary Figure 5 | Energy Dispersive X-ray (EDX) element maps for sample CCVP. First panel shows layered image, subsequent panels are broken down by element.

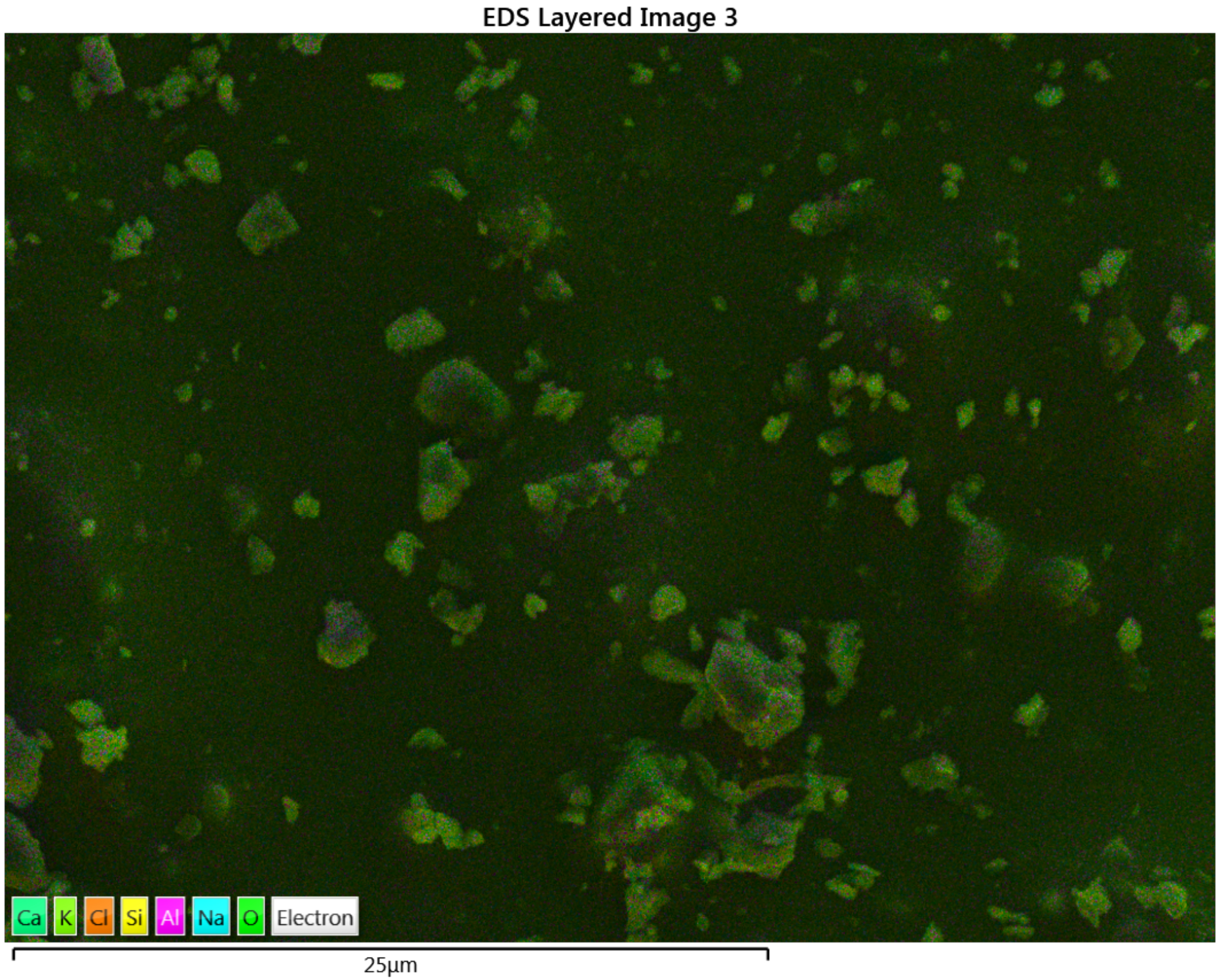

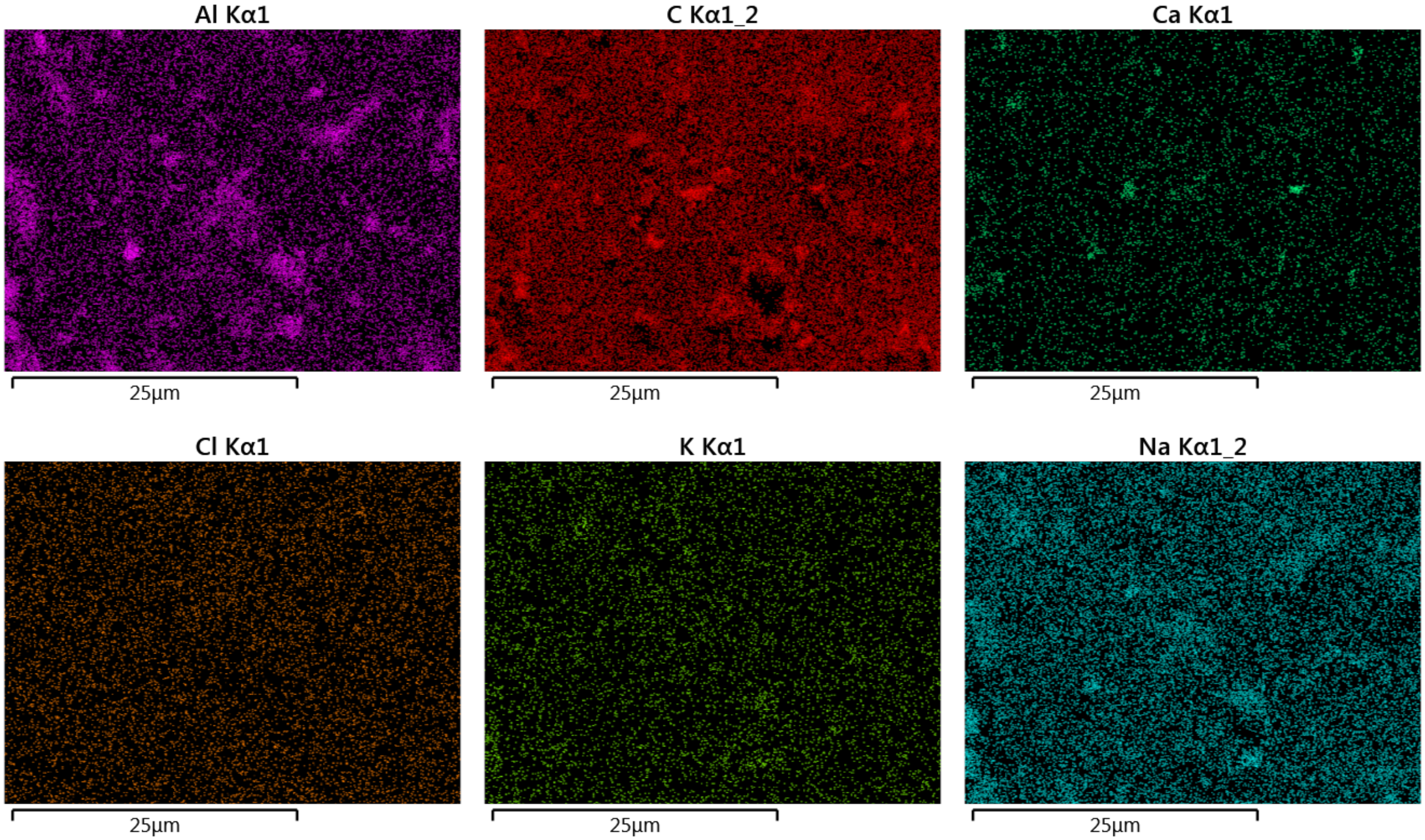
Al Kα1
C Kα1_2
Ca Kα1
Cl Kα1
K Kα1
Na Kα1_2
25μm
25μm
25μm
25μm
25μm
25μm

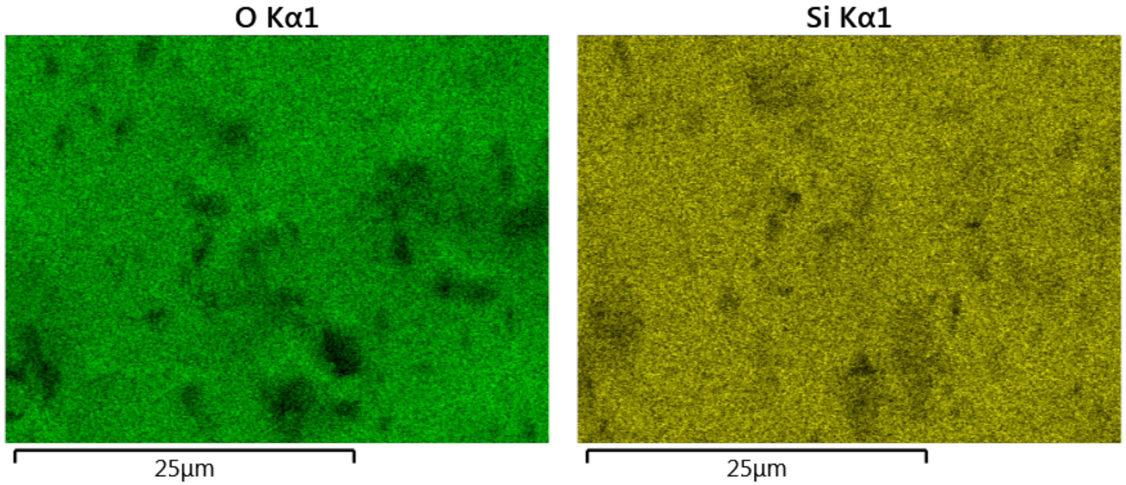
O Kα1
Si Kα1
25μm
25μm
